# A tripartite model system for Southern Ocean diatom-bacterial interactions reveals the coexistence of competing symbiotic strategies

**DOI:** 10.1038/s43705-022-00181-w

**Published:** 2022-10-03

**Authors:** Sarah Andrew, Travis Wilson, Stephanie Smith, Adrian Marchetti, Alecia N. Septer

**Affiliations:** 1 Department of Earth, Marine & Environmental Sciences, University of North Carolina, Chapel Hill, NC USA; 2 Environment, Ecology & Energy Program, University of North Carolina, Chapel Hill, NC USA

**Keywords:** Biogeochemistry, Water microbiology, Microbial ecology, Marine microbiology

## Abstract

Southern Ocean (SO) diatoms play an important role in global carbon flux, and their influence on carbon export is directly linked to interactions with epiphytic bacteria. Bacterial symbionts that increase diatom growth promote atmospheric carbon uptake, while bacterial degraders divert diatom biomass into the microbial loop where it can then be released as carbon dioxide through respiration. To further explore SO diatom-bacterial associations, a natural model system is needed that is representative of these diverse and important interactions. Here, we use concurrent cultivation to isolate a species of the ecologically-important SO diatom, *Pseudo-nitzschia subcurvata*, and its co-occurring bacteria. Although vitamin-depleted, axenic *Pseudo-nitzschia* grew poorly in culture, addition of a co-isolated Roseobacter promoted diatom growth, while addition of a co-isolated Flavobacterium negatively impacted diatom growth. Microscopy revealed both bacterial isolates are physically associated with diatom cells and genome sequencing identified important predicted functions including vitamin synthesis, motility, cell attachment mechanisms, and diverse antimicrobial weapons that could be used for interbacterial competition. These findings revealed the natural coexistence of competing symbiotic strategies of diatom-associated bacteria in the SO, and the utility of this tripartite system, composed of a diatom and two bacterial strains, as a co-culture model to probe ecological-relevant interactions between diatoms and the bacteria that compete for access to the phycosphere.

## Introduction

Single-celled photosynthetic eukaryotes, particularly diatoms, are the main primary producers in polar oceans, forming massive annual blooms and comprising the base of the polar food chain [1]. Although these organisms are microscopic, their collective numbers are sufficient to influence the biogeochemistry of the planet, supplying a significant fraction of the Earth’s oxygen [2]. Marine bacteria are also highly abundant in seawater [3], playing important roles in assimilating and decomposing a significant portion of the organic carbon fixed by diatoms [4–8]. Together, phototrophic eukaryotes and heterotrophic bacteria constitute components of the “microbial loop”, where phytoplankton and bacteria contribute significantly to the cycling of carbon and other important nutrients [9]. Furthermore, bacteria are known to directly compete with phytoplankton for resources, such as iron (Fe); and resource availability (light, dissolved organic carbon, Fe) have been modelled to directly regulate ecological phytoplankton-bacterial interactions [10]. Emerging work has also revealed that important symbiotic relationships (i.e., “the living together of differently named organisms” [11]) exist between phytoplankton and heterotrophic bacterial cells [8, 12]. For example, many diatoms cannot synthesize essential vitamins or detoxify byproducts from their own metabolism and require the help of specific symbiotic bacteria to fulfill these roles [13, 14]. In turn, the bacterial partners receive organic carbon and other nutrients excreted from diatom cells that support bacterial growth [15]. Thus, these mutually-beneficial symbiotic relationships, or mutualisms, allow both phytoplankton and marine bacteria to flourish in an otherwise harsh environment.

Some bacterial species can colonize the phycosphere, the diffusive boundary layer around individual diatom cells that is rich in the organic and inorganic compounds released by the diatom [8, 16]. Indeed, several studies characterizing the prevalence and diversity of bacteria attached to diatoms in situ found the proportion of phytoplankton cells with attached bacteria varied widely (5–80%) with abundance of attached bacteria ranging from 1–61 bacterial cells per diatom [17, 18]. The attached microbiome for a given cell contained between one and eleven bacterial phylotypes [17], and others showed that the relative abundance of attached bacterial phylotypes changed significantly with the growth state of the diatom host and nutrient availability [19]. Taken together, these studies suggest that phytoplankton commonly have attached bacteria and these associations can by influenced by both biotic and abiotic factors.

Because the symbiotic relationship between phytoplankton and bacteria, which can be mutualistic or parasitic, is built around the chemicals exchanged between partners [5, 8], mechanisms have evolved to promote select partner matching to favor mutual benefits [20]. For example, certain marine bacteria preferentially swim toward the unique chemical cocktail released by specific phytoplankton species [16]. Once the association is established, both partners are thought to have evolved strategies to maintain close physical contact: diatoms may retain bacterial symbionts in excreted mucus [21], or bacteria may use surface-exposed proteins to adhere directly to the phytoplankton cell [22]. Results from in situ characterization and coculture approaches have begun to dissect the specifics of these interactions and revealed that bacteria likely compete for access to the phycosphere, with the winner of this interbacterial competition having vastly different effects on the phytoplankton cell, based on their ecological role as a growth promoter (i.e., mutualistic) or a degrader (i.e., parasitic) [8]. For example, diatom-associated bacteria are known to provide nutrients (eg. vitamins, Fe and NH_4_) [5, 6], deter invasion by other opportunistic bacteria [23, 24], or directly lyse/degrade diatom cells [25].

Although much of the past work on phytoplankton-bacterial interactions has focused on temperate regions, these symbiotic associations are also critical in polar habitats, such as the Southern Ocean (SO). The SO accounts for 40% of anthropogenic CO_2_ uptake [26], but phytoplankton growth here is often limited by low Fe availability, with seasonal colimitation by light [27, 28]. Additional evidence shows that diatom blooms are often B vitamin (e.g., B_1_, B_7_ and B_12_) limited, due to the roles these vitamins play as co-factors in essential enzymes [29]. Moreover, B vitamins are biologically derived, and therefore can be growth limiting to organisms that cannot synthesize their own B vitamins, relying on an external biological source for their own needs [30]. *Pseudo-nitzschia* is a genus of pennate diatom encompassing over 50 species that are globally distributed [31] and numerically abundant in the SO, where it comprises 13–71% of diatoms in the Weddell Sea [32–35]. Examined members of this genus have an obligate requirement for cobalamin (Vitamin B_12_) as a co-factor in the methionine synthase enzyme (MetH), and do not contain the cobalamin-independent methionine synthase (MetE) [36], suggesting that the diatom obtains cobalamin externally, likely through interactions with B_12_-producing bacteria. However, in order to directly test such hypotheses, lab-based experiments would be preferred over those in situ because a lab setting would allow for better control of variables such as the presence of specific phytoplankton and bacterial species, as well as abundance of macronutrients, micronutrients, and trace metals.

Despite the broad ecological importance of SO diatoms, we are lacking a natural, co-evolved culture-based model system that is representative of complex diatom-bacterial interactions that can be interrogated in the lab. Particularly for bacterial-diatom interactions that are close physical associations. Such a coculture model system would allow researchers to directly test in the lab hypotheses generated from in situ observations and even develop predictions as to how future climate conditions might impact these critical symbiotic associations and their contributions to nutrient cycling, oxygen production, and carbon export. To fill this knowledge gap, we reasoned that if growth promoters and degraders are physically associated with SO diatoms, we could design an approach to concurrently cultivate both partners in isolation and add them back together to determine the fate of the interactions. To this end, we identified four bacteria that are closely associated with the SO diatom *Pseudo-nitzschia subcurvata*, two of which have positive or negative growth effects under multi-vitamin-depleted conditions. Finally, the genomes of these bacterial isolates reveal insights into their metabolic strategies that may be coordinated by light-responsive transcriptional regulators and indicate their predicted capabilities to kill bacterial competitors.

## Methods

### Diatom isolation and culturing conditions

**Fig. 1:**
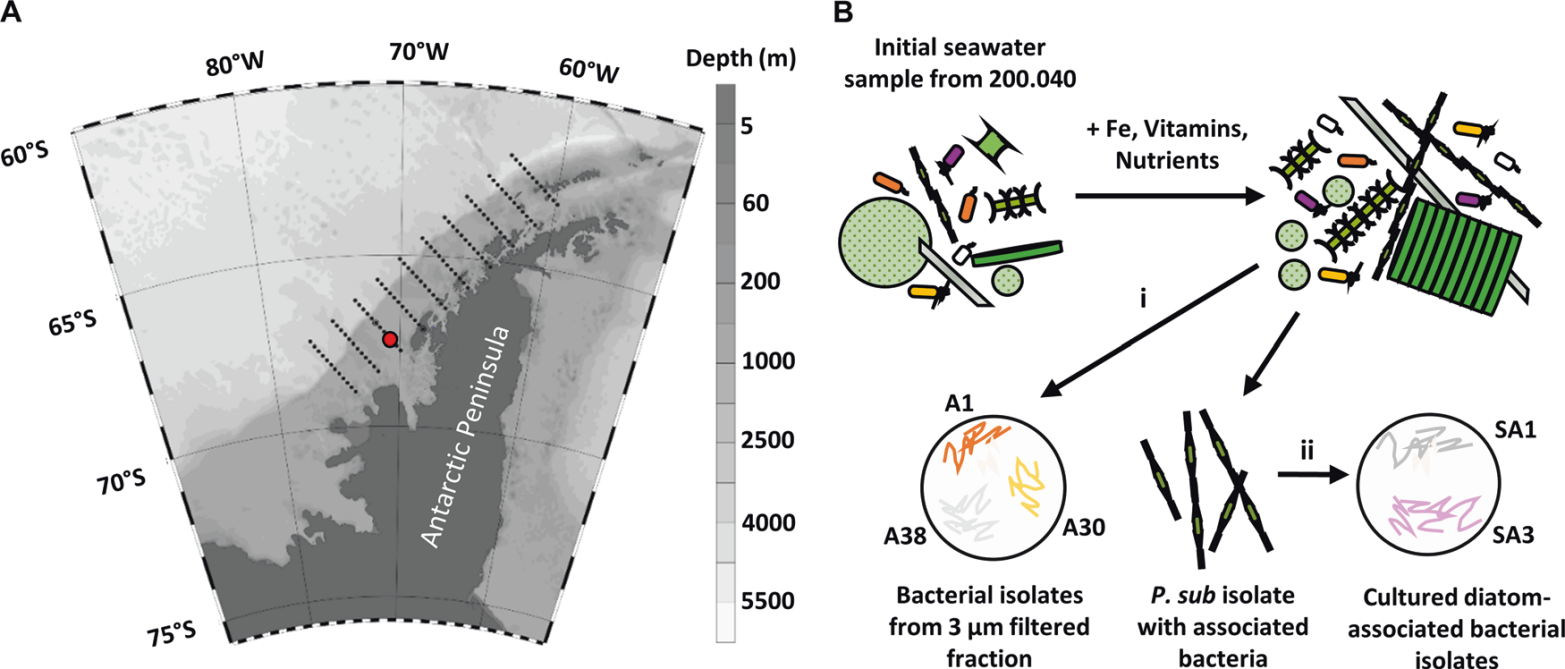
Bacterial and diatom cultures were coisolated from the same SO water sample. **A** Map of isolation location. Black parallel dotted lines indicate LTER sampling grid at Palmer Station. Red circle is 200.040 where seawater was collected at the surface. Gradient is bathymetry in meters. **B** Schematic of cultivation and isolation of *P. subcurvata* and bacterial isolates from (i) 3 um fraction of phytoplankton enrichment culture, or (ii) closely associated with monoclonal *P. subcurvata* culture after passage for 12 months.

Monoclonal diatom and bacterial isolates were established from seawater taken from the Western Antarctic Peninsula along the Palmer LTER sampling grid (67°51.02′S, 70°58.96′W) in January 2019 (Fig. 1A). Seawater samples were immediately enriched with vitamins, trace metals, and macronutrients, and kept at 2.5 °C and ~150 photons m^−2^ s^−1^. A subsample of the initial seawater sample was transferred into the artificial seawater medium, Aquil [37], and the diatom *Pseudo-nitzschia subcurvata* was isolated using serial dilutions into Aquil by light microscopy (40x magnification, Olympus CKX41) with a sterile micropipette (Fig. 1B). *Pseudo-nitzschia subcurvata* (strain UNC1901) was identified by PCR amplification and sequencing of the 18S-ITS1-5.8S region [38]. Briefly, DNA was extracted from algal pellets using DNeasy Plant Mini Kit (Qiagen), according to the manufacturer instructions. PCR was performed using primers selected to amplify the 18S-ITS1 region. PCR products were purified using QIAquick PCR Purification Kit (Qiagen) and sequenced by Sanger DNA sequencing (Genewiz). Sequences were aligned using MUSCLE [39] and BLASTn sequence homology searches were performed against the NCBI nucleotide nonredundant (nr) database to determine species with a cutoff identity of 98% [40].

Experimental diatom cultures were maintained under continuous illumination (100 ± 10 µmol photons m^−2^ s^−1^) in 28 mL polycarbonate tubes at 4 °C as semi-continuous batch cultures in trace metal ion buffered artificial seawater medium Aquil [37]. Aquil medium was microwave-sterilized and enriched with filter-sterilized (0.2 μm, Gelman Acrodisc PF) trace metals, and macronutrients containing 300 μmol L^−1^ nitrate, 100 μmol L^−1^ silicic acid, and 10 μmol L^−1^ phosphate, supplemented with 1370 nM filter-sterilized FeCl_3_ in the presence of 100 μmol L^−1^ EDTA. Growth-replete vitamin treatments (+vitamins) were enriched with a filter-sterilized vitamin cocktail: 297 nmol L^−1^ Thiamin (B_1_), 2047 pmol L^−1^ Biotin (B_7_), 348 pmol L^−1^ Cobalamin (B_12_) [37]. In vitamin-deplete treatments (-vitamins) all vitamins (B_1_, B_7_, B_12_) were excluded from the growth media. Diatom growth was monitored by *in vivo* Chlorophyll *a* fluorescence using a Turner Designs model 10-AU [41].

### Bacterial isolation

To isolate the closely associated bacteria, SO phytoplankton enrichment cultures were applied to a vacuum filtration system fitted with a 3 µm filter. The filter was then rinsed with sterile 20 g L^−1^ NaCl to rinse loosely-associated bacteria from phytoplankton cells and the vacuum was applied. Next, the filter was removed from the housing and placed into a 50 mL conical tube with 5 mL of NaCl solution and vortexed to re-suspend phytoplankton and their associated bacteria. 50 µL of filtered culture were spread onto 1/10 YTSS plates: [20 g L^−1^ Instant Ocean Salts, 0.5 g L^−1^ Tryptone, 0.8 g L^−1^ Yeast Extract, 15 g L^−1^ agar]. The remaining filtered culture from the 50 mL conical tube was poured into a filter housing containing a 0.2 µm filter and the vacuum was applied to capture all remaining bacteria. The 0.2 µm filter was placed into a 1/10 YTSS plate. Inoculated plates were incubated at 4 °C and 10–12 °C for 73 days and were checked for new growth weekly. Colonies were collected and re-streaked onto 1/10 YTSS plates three times to ensure the strain was isolated (Fig. 1B).

Additional bacteria associated with the diatom *P. subcurvata* were isolated from one year old diatom monocultures (Table S1). Briefly, 30 mL of *P. subcurvata* were filtered onto sterile 5 µm polycarbonate filters (25 mm, Isopore) from exponentially growing cultures. Filters were streaked onto 1/10 YTSS agar plates and incubated at 4 °C for 2–4 weeks. The plates were examined for bacterial growth at 5-day intervals over 4 weeks. Colonies were transferred onto 1/10 YTSS plates, single colonies picked and then restreaked on to fresh medium at least twice or until pure.

### 16S sequencing and phylogenetic tree

The 16S rRNA gene was PCR-amplified using Econotaq and primers 8F (5′-AGAGTTTGATCCTGGCTCAG-3′) and 1492r primer (5′-TACCTTGTTACGACTT). The size of the PCR products was determined using gel electrophoresis, then cleaned using DNA Clean & Concentrator-5 Kit (Zymo), and sequenced using Eton Bioscience, Inc. 16S sequences were aligned using MUSCLE [39] with default parameters, and trimmed in Jalview v2.11.1.6 [42]. Publicly available 16S sequences were obtained from the NCBI database as references. A maximum likelihood tree was constructed using the IQtree webserver [43], using the best-fit model K2P + G4 automatically chosen according to Bayesian Information Criterion by ModelFinder [44, 45], with 1,000 bootstraps [46]. The tree was visualized using the Interactive Tree of Life (iTOL) v.4 webserver (https://itol.embl.de/) [47].

### Genome sequencing of bacterial isolates

Genomic DNA was extracted from bacterial biomass grown on 1/10 YTSS plates using the Zymo Quick-DNA Fungal/Bacterial Kit (Irvine, CA) following manufacturer protocols. Concentration and purity of DNA were measured and recorded using a BioSpectrometer Basic (Eppendorf). Whole genome sequencing was performed using paired end (2 × 150 bp) reads on the Illumina NextSeq 550 to a depth of 150 Mbases at the Microbial Genome Sequencing Center (MiGS, Pittsburgh, PA). A total of 2.6 M and 3.8 M raw, paired-end reads were sequenced for isolates A30 and SA1, respectively. Raw reads were trimmed using BBDuk v.38.84 with a leading/trailing minimum quality of 6, and minimum read length of 10. Quality trimmed reads were assembled using SPAdes v.3.13.0 with default settings [48]. Genome completion estimates were determined using the Microbial Genomes Atlas (MiGA) [49]. Average nucleotide identity (ANI) was calculated using FastANI [50]. Bacterial genomes were annotated using Prokka v.1.14.6 using a minimum contig length of 200 [51]. The Whole Genome Shotgun project and raw reads are deposited at DDBJ/ENA/GenBank under the BioSample accession IDs SAMN25067897 (A30) and SAMN25067898 (SA1).

### Establishment of *P. subcurvata* axenic culture

*The diatom P. subcurvata* was rendered axenic using methods described previously [36]. In brief, 5 mL of a dense exponentially growing culture was incubated for 24 h with an antibiotic cocktail containing 50 μg mL^−1^ streptomycin, 66.6 μg mL^−1^ gentamycin, 20 μg mL^−1^ ciprofloxacin, 2.2 μg mL^−1^ chloramphincol (in ethanol), and 100 μg mL^−1^ ampicillin. A 1 mL aliquot was transferred into sterile, antibiotic free, Aquil medium and incubated under the light and temperature conditions described above. Subsequently, the entire process was repeated two more times to ensure bacterial abundances were substantially reduced. Cultures were checked periodically for bacterial contamination by checking for bacterial growth in ASWT (artificial seawater-tryptone) medium (per liter: 700 mL Instant Ocean at 35 ppt, 300 mL DI water, 5g tryptone, 3 g yeast extract, 3 mL 50% glycerol), in addition to Sybr Green I (Invitrogen) and DAPI (Thermofisher) staining and visualization with an Olympus BX61 epifluorescence microscope [52]. The resultant antibiotic-treated *P. subcurvata* culture was left to acclimate to no bacteria for approximately 100 generations and was subsequently used for the bacteria coculture experiment.

### Vitamin-deplete Bacteria —diatom coculture experiments

Triplicate vitamin-depleted diatom treatments were prepared by inoculating vitamin-free Aquil with exponentially growing vitamin-replete antibiotic-treated *P. subcurvata* cultures in a 1:100 dilution. All cultures were grown at the light and temperature settings described above. Cultures were grown until mid-exponential phase growth and transferred to fresh media in a 1:100 dilution to maintain diatoms in the exponential growth phase. These transfers were repeated until cellular vitamin stores were depleted, and growth of the vitamin-limited cultures no longer grew exponentially. After vitamin-limited diatoms were maintained in stationary phase for four days, each set of triplicate antibiotic-treated, vitamin-limited *P. subcurvata* cultures were used to receive different bacterial strain additions. Diatom growth was determined by measuring RFU over time for *P. subcurvata* grown alone or with indicated bacterial strains added at day 0 at ~10^4^–10^5^ colony forming units (CFUs) per mL. Bacterial isolates include: *Sulfitobacter* sp. SA1, *Olleya* sp. A30, *Pseudoalteromonas* sp. A1, *Colwellia* sp. A38, and *Glaciecola* sp. SA3. Final bacterial abundance (CFU/ml) was determined for indicated bacteria grown alone or with *P. subcurvata* at the end of the coculture experiment (43 days) by plating dilution series of cocultures onto 1/10th YTSS agar plates and incubating at 10 °C until colonies became visible for counting (usually 5–10 days).

## Results

### Co-isolation of an Antarctic diatom and associated bacterial strains from the Southern Ocean

**Fig. 2:**
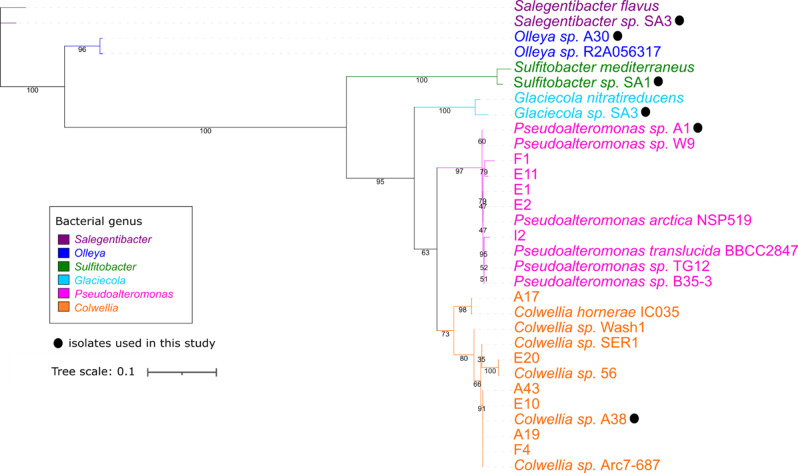
Phylogenetic characterization of bacterial isolates. Phylogenetic tree based on 16S rRNA DNA sequences of indicated reference strains and bacterial isolates. Clades are color-coded by bacterial genera and isolates used for cocultures are indicated by a black circle. Class-level designation is as follows: Flavobacteria (purple and blue), Alphaproteobacteria (green), Gammaproteobacteria (cyan, pink, orange). Node values represent maximum likelihood bootstrap.

To obtain isolates of naturally co-occurring diatom and bacteria, we collected seawater samples from the Southern Ocean near Palmer Station (Fig. 1A). The initial seawater sample was enriched for phytoplankton growth by amending samples with a nutrient cocktail, then maintained under ambient surface seawater conditions. From this enrichment a pennate diatom was obtained and verified to be of the species *Pseudo-nitzschia subcurvata*. Bacterial isolates were cultivated in two ways: directly from the 3 µm fraction of the initial phytoplankton enrichment culture, as well as directly from the *P. subcurvata* monoculture established after successful isolation from other phytoplankton species (Fig. 1B). Bacterial isolates were genotyped based on their 16S rRNA gene sequence and include species from the following genera: *Sulfitobacter*, *Pseudoalteromonas*, *Colwellia*, *Olleya*, and *Glaciecola*. These heterotrophic bacterial isolates are representative of what has previously been described for high-latitude and/or phytoplankton-associated bacteria [8, 53, 54], which include members of the Roseobacter and Flavobacteriaceae clades (Fig. 2 and Table S1).

### Xenic *P. subcurvata* cultures survive longer than antibiotic-treated cultures

**Fig. 3:**
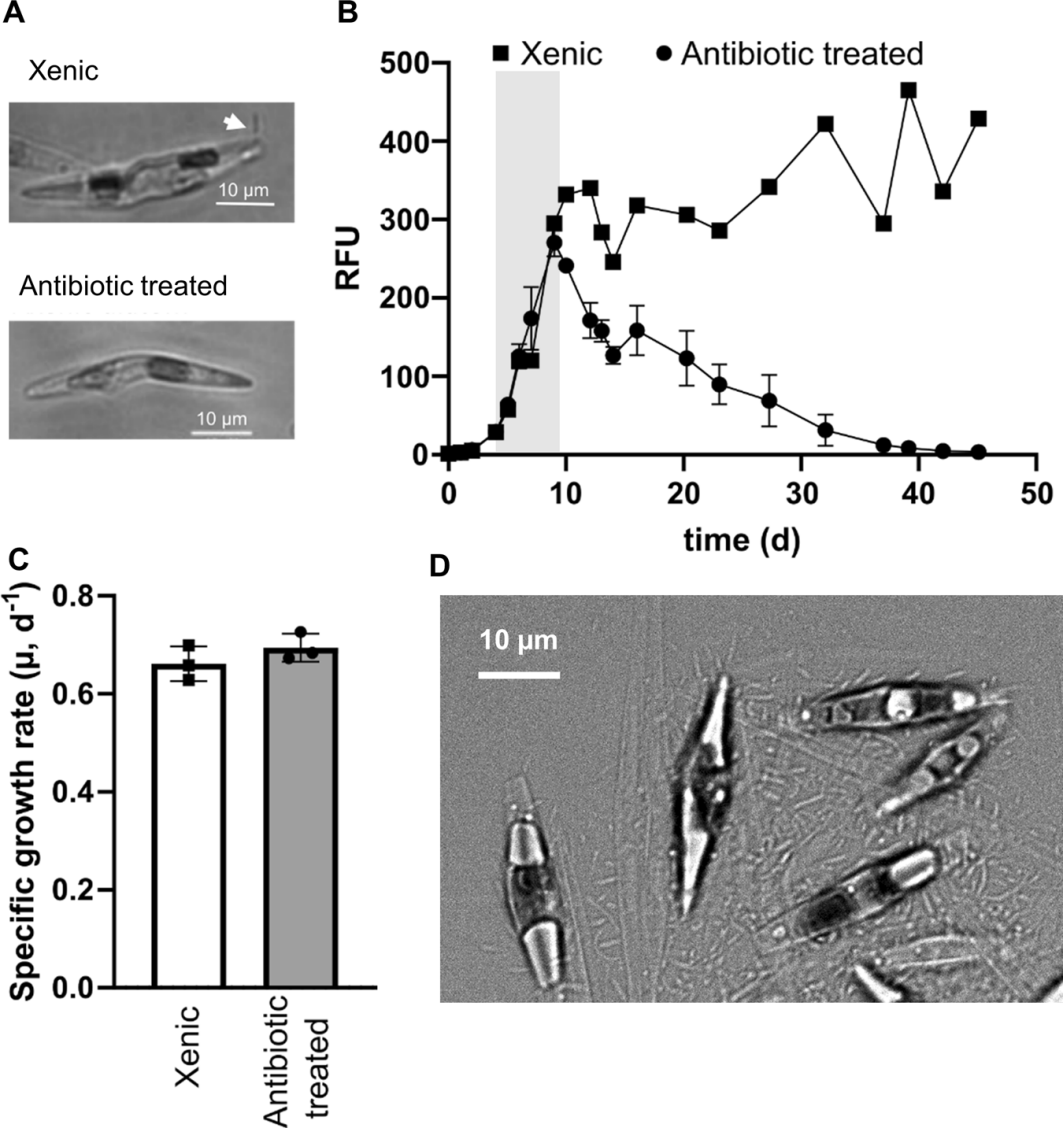
Co-isolated bacterial community promotes stationary phase survival for *P. subcurvata* grown in nutrient replete conditions. **A** DIC microscopy images of *P. subcurvata* cells from xenic cultures (top) or after treatment with antibiotic cocktail (bottom). Arrow indicates bacterial epibiont. **B** Relative Fluorescence Units (RFU) as a proxy for diatom growth over time for xenic (squares) and antibiotic-treated (circles) *P. subcurvata* culture grown under nutrient-replete conditions. Gray shading indicates time points (days 4–9) used to calculate growth rates in **C**. **C** Growth rates of *P. subcurvata* calculated during exponential phase growth. Error bars are the standard deviation of 3 biological replicates. **D** DIC microscopy image of xenic *P. subcurvata* cells covered with epibionts after growth in nutrient-replete conditions for ~90 days.

Although our *P. subcurvata* culture contains only one species of phytoplankton, it carried with it native bacteria from the initial enrichment culture inoculum, originating from the Southern Ocean. We routinely observed bacteria adhered at the cell poles to *P. subcurvata* cells in the xenic cultures (Fig. 3A). However, after *P. subcurvata* cultures were exposed to an antibiotic cocktail, we no longer detected the presence of bacterial cells by culture-dependent methods or microscopy (Fig. 3A). Given the importance of diatom symbionts described above, we predicted that the xenic *P. subcurvata* cultures may grow or survive better than the antibiotic-treated cultures. To test this prediction, xenic and antibiotic-treated *P. subcurvata* were grown in vitamin-replete media over 50 days (Fig. 3B). The exponentially growing antibiotic-treated and xenic *P. subcurvata* cultures grew equally well until stationary growth was reached on day 9–10 (Fig. 3B, C). In comparison with the xenic *P. subcurvata* culture, the RFU of the antibiotic-treated culture declined over the following 30 days. Moreover, when we imaged *P. subcurvata* cells from late stationary xenic cultures (~3 months), we observed diatom cells covered in rod-shaped bacteria (Fig. 3D). Taken together, these data indicate that *P. subcurvata* relies on bacterial symbionts to promote diatom survival in stationary phase.

### Vitamin-depleted co-culture experiments of antibiotic-treated *P. subcurvata* and bacterial isolates

**Fig. 4:**
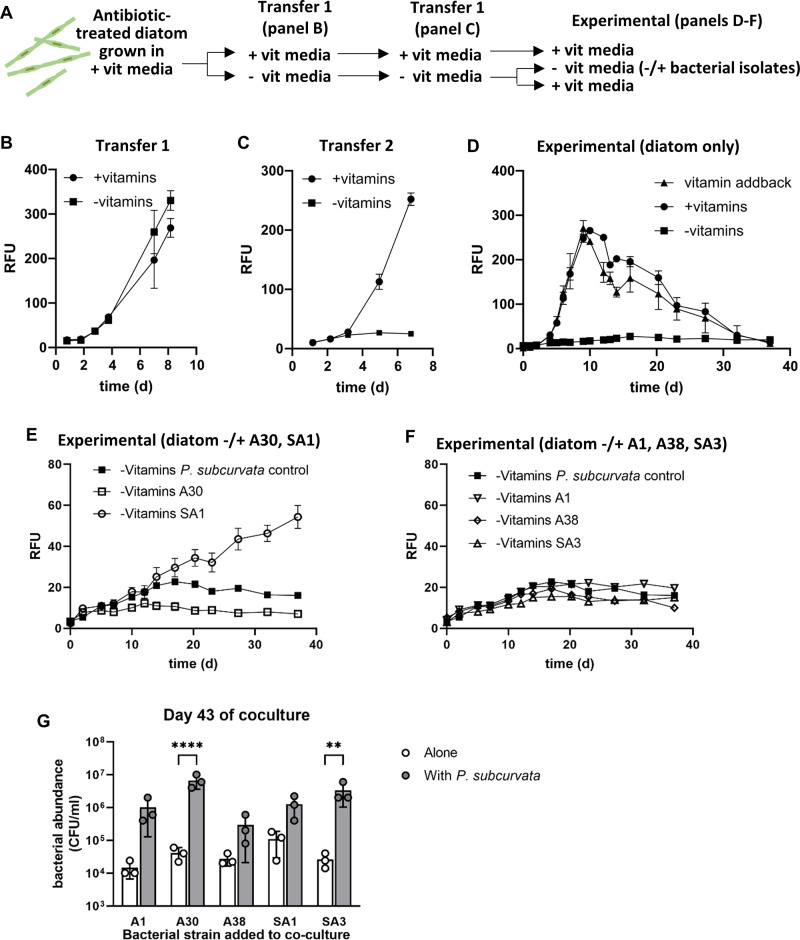
Effects of bacterial isolates on diatom growth under vitamin-limited conditions. **A** Diagram outlining experimental method used to obtain vitamin-depleted diatom cultures and subsequent culture experiment without and with added vitamins or bacterial isolates. **B**-**D** RFU values over time for antibiotic-treated *P. subcurvata* cultures to achieve vitamin-depleted cultures by serial transfer into fresh media (day 0) without (squares) or with (circles) added vitamins (297 nmol L^−1^ Thiamin (B1), 2047 pmol L^−1^ Biotin (B7), 348 pmol L^−1^ Cobalamin (B12)). Error bars are the standard deviation of three biological replicates. **B** Transfer 1 of culture in vitamin replete or vitamin deplete media. **C** Serial transfer from panel **B** at Day 8 of culture into new medium, showing vitamin limitation from Days 3–4. **D** Serial transfer of culture into new media from panel **C** at Day 7, including the growth response of vitamin-depleted cultures (-vitamins, squares) when transferred into vitamin-replete media (vitamin addback, triangles). **E**, **F** RFU over time for antibiotic-treated, vitamin-limited *P. subcurvata* grown alone (filled square) or with indicated bacterial strains added at day 0 at ~10^4^–10^5^ colony forming units (CFUs) per mL. Bacterial isolates include: *Sulfitobacter* sp. SA1 (open circles), *Olleya* sp. A30 (open squares), *Pseudoalteromonas* sp. A1 (inverted open triangles), *Colwellia* sp. A38 (open diamonds), and *Glaciecola* sp. SA3 (open triangles). **G** Bacterial abundance in CFU/ml for indicated bacteria grown alone (white) or with *P. subcurvata* (gray) at the end of the coculture experiment (43 days). Error bars indicate standard error. Asterisks indicate statistical significance between values for bacterial cultures alone vs in coculture: 2way ANOVA, uncorrected Fisher’s LSD, **** indicates *p* < 0.0001, ** indicates *p* < 0.005.

We next used a coculture approach to determine whether any of the bacterial isolates we cultured might influence *P. subcurvata* growth under B vitamin-depleted conditions (Fig. 4A). We predicted three possible outcomes for diatom growth with bacterial addition: (i) diatom growth is enhanced, (ii) diatom growth is unchanged, or (iii) diatom growth declines. However, because the cocultured heterotrophic bacteria use organic carbon from diatoms regardless of the nature of the interaction (growth promoter or degrader), we predicted the growth of all bacterial strains would increase in coculture with the diatom, relative to growing alone in culture medium lacking a source of organic carbon.

Depleting *P. subcurvata* of B vitamins required several transfers in vitamin-free media in order to deplete internal stores. Specifically, exponentially growing antibiotic-treated vitamin-replete diatom cultures were transferred to vitamin-free media, and growth was assessed by measuring RFU over time. The vitamin-free treatment culture grew just as well as the vitamin-replete control culture (Fig. 4B), suggesting additional transfers in vitamin-free media would be required to deplete *P. subcurvata* of internal vitamin stores. A second transfer resulted in no growth in the vitamin-free treatment (Fig. 4C). To ensure the lack of growth was due to vitamin depletion and not colimitation of another nutrient, cultures were again transferred into fresh media, including transferring the vitamin-depleted culture into vitamin-replete media. Both treatments with vitamins grew equally well (Fig. 4D), indicating two transfers in vitamin-free media resulted in vitamin-depleted *P. subcurvata* cultures that could be rescued by adding back vitamins.

To determine the impact of co-isolated bacteria on diatom growth, antibiotic-treated, vitamin-depleted cultures of *P. subcurvata* were inoculated with five individual bacterial isolates: *Sulfitobacter* sp. SA1, *Olleya* sp. A30, *Pseudoalteromonas* sp. A1, *Colwellia* sp. A38, and *Glaciecola* sp. SA3 (Fig. 4A). The growth of vitamin-depleted *P. subcurvata* differed depending on the bacterial addition. The *P. subcurvata* cultures inoculated with *Sulfitobacter* sp. SA1 grew significantly better than the *P. subcurvata* control with no bacteria added, but co-cultures inoculated with *Olleya* sp. A30 had lower RFU values than the *P. subcurvata* control, however there was no significant difference in RFU by the end of the experiment (Fig. 4E, Fig S1). In contrast, the RFU values of *P. subcurvata* co-cultures inoculated with *Pseudoalteromonas* sp. A1, *Colwellia* sp. A38 or *Glaciecola* sp. SA3 were similar to the axenic control throughout. (Fig. 4F, Fig. S1). Moreover, the calculated growth rates for *P. subcurvata* in coculture with *Sulfitobacter* sp. SA1 (0.03 ± 0.00 d^−1^) were ~4% of the growth rates for the vitamin addition control (0.69 ± 0.02 d^−1^), indicating that diatom cells in coculture with SA1 were vitamin-limited rather than vitamin-depleted. In comparison, the vitamin-depleted diatom cells grew at −0.01 ± 0.00 d^−1^ over the same time period (Fig. S2). At the termination of the experiment, bacterial abundance was >10-fold higher in co-culture with *P. subcurvata*, rather than in media alone, which lacked a source of organic carbon, but this relationship was only statistically significant for A30 and SA3 (Fig. 4G).

**Fig. 5:**
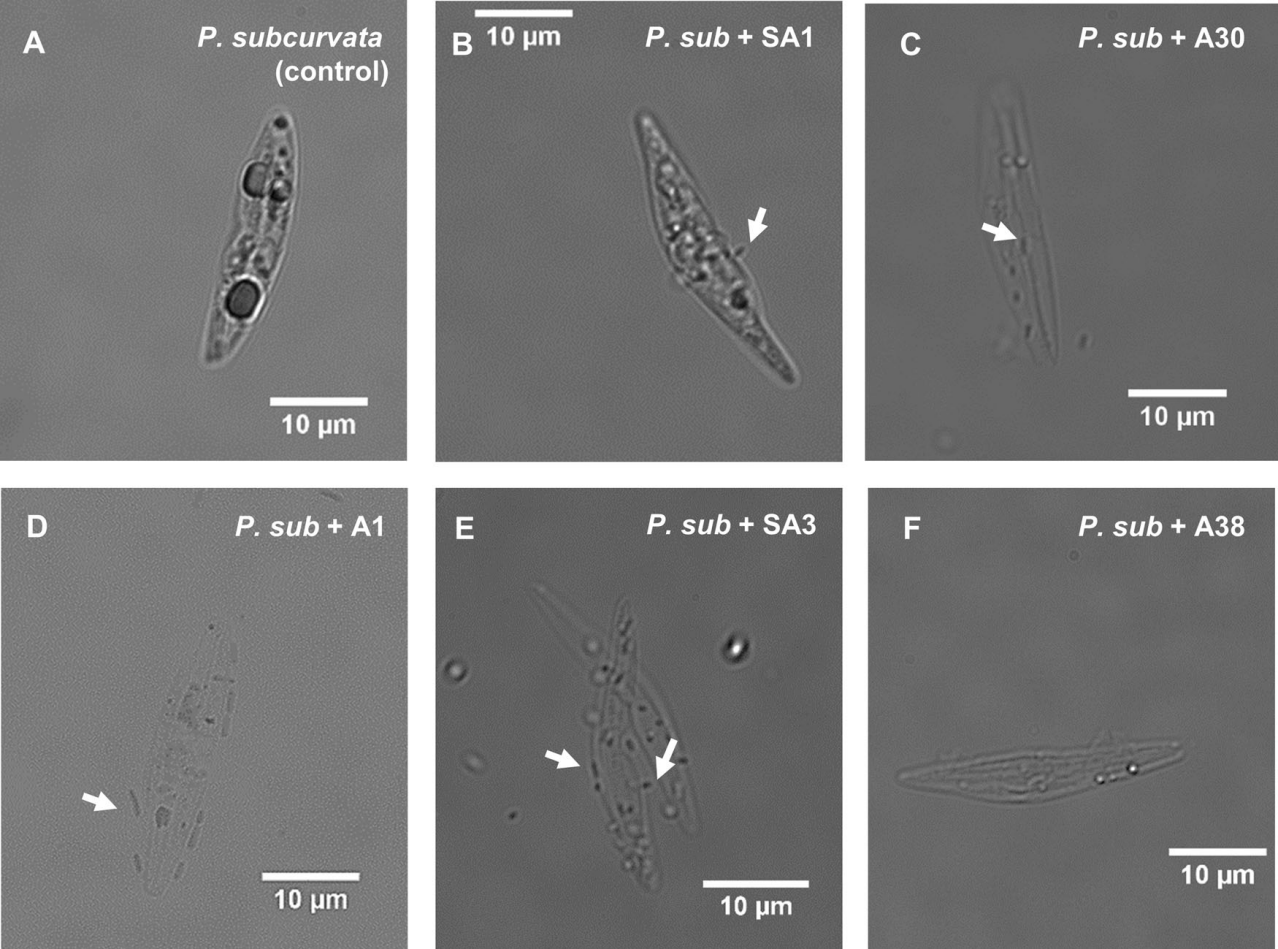
Co-isolated bacterial strains physically associate with *P. subcurvata* in coculture. Single cell DIC microscopy images were taken with a Nikon Ti-2 inverted epifluorescence microscope using 100x objective and NIS Elements acquisition software. Images are from vitamin-limited cocultures at day 42. Images are representatives of *P. subcurvata* cells grown alone (**A**) or with indicated bacteria (**B**–**F**). Arrows indicate bacterial cells.

Microscopic analysis of the co-cultures revealed diverse strategies for physical association between the bacteria and diatom (Fig. 5). Although no cells were seen associated with the antibiotic-treated *P. subcurvata* control (Fig. 5A), *Sulfitobacter* sp. SA1 cells were observed to physically associate with *P. subcurvata* by attaching at the poles of the bacterium (Fig. 5B). In comparison, *Olleya* sp. A30 cells were observed to occur inside of empty *P. subcurvata* frustules (Fig. 5C). *Pseudoalteromonas* sp. A1 cells were observed to physically associate with the frustule of *P. subcurvata* along the length of the bacterium (Fig. 5D). Bacterial cells in the *Glaciecola* sp. SA3 coculture were seen attached either at their poles or along their length (Fig. 5E). Meanwhile, no physical associations were observed between *Colwellia* sp. A38 cells and *P. subcurvata* (Fig. 5F).

### Bacterial genomes reveal strategies for symbiotic interactions

To determine the functional potential of some of our bacterial isolates, we used whole genome sequencing of a representative of each type of interaction by selecting strains *Sulfitobacter* sp. SA1 (positive interaction), *Glaciecola* sp. SA3 (neutral interaction), and *Olleya* sp. A30 (negative interaction). The sequence analysis indicated that *Sulfitobacter* sp. SA1 and *Olleya* sp. A30 draft genomes are high quality with estimated sizes of ~3.8 and ~3.7 Mbp, respectively, encoding >3400 genes each (Table 1). However, analysis of the SA3 sequences indicated it was not a pure culture of *Glaciecola*, as we expected from 16S gene sequencing results, but rather a mix of *Glaciecola sp*. and *Salegentibacter* sp. The fact that SA3 is actually a mix of two species could explain why we saw two modes of cell attachment in our coculture microscopy images: bacterial cells attached at the poles or along their side (Fig. 5E). Therefore, because SA3 sequences were a mix of two strains, we chose to further analyze only the genomes of the two pure cultures: *Sulfitobacter* sp. SA1 and *Olleya* sp. A30.

**Table 1:** Genome statistics for draft genomes of *Sulfitobacter* sp. SA1 and *Olleya* sp. A30.

Genome statistics	SA1	A30
# of contigs >1000 bp	50	42
% completeness	100%	98.60%
% redundancy	1.41	0
Estimated size (Mb)	3.805	3.684
# of predicted genes	3831	3429

Assembly statistics were acquired using SPAdes and gene number estimates were made using prokka.

The functional predictions for B and other vitamin biosynthesis correlated with the results of our coculture experiments (Table 2). The genome of strain SA1, which enhanced *P. subcurvata* growth under vitamin-depleted conditions (Fig. 4E), encodes predicted biosynthetic pathways for cobalamin (B_12_), folate, thiamine (B_1_), and phylloquinone (K_1_). However, for strain A30, which caused a decrease in RFU, functional predictions suggest that although A30 may be able to synthesize folate, thiamine, and biotin (B_7_), it appears to lack the B_12_ biosynthetic pathway and instead, encodes B_12_ transporters (Table 2). Given that *P. subcurvata* is auxotrophic for B_12_ vitamin, these findings provide one possible explanation for why SA1, but not A30, promotes diatom growth in the absence of Vitamin B_12_.

**Table 2:** Predicted functional genes for *Sulfitobacter sp*. SA1 and *Olleya sp*. A30.

Predicted function	SA1	A30		Predicted function	SA1	A30
*Vitamin biosynthesis or transport*				*Iron acquisition*		
biotin biosynthesis	+	+		iron hydroxamate transport	+	
Vitamin B12 biosynthesis	+			iron ABC-type transport	+	
Vitamin B12 transporters		+		FepD siderophore transport	+	
folate biosynthesis	+	+		Bacterioferritin-associated ferrodoxin Bfd	+	
thiamine biosynthesis	+	+		ferritin-like metal binding protein YciE	+	
phylloquinone (Vitamin K_1_) biosynthesis	+			FebA/FepB siderophore transport		+
				Iron transporters FeoA/CirA	+	
*AA and Carbohydrate Transport/Metab*.	313	153		ferric dicitrate transport (FecR)		+
proteases	26	17				
Spermidine*/putrescine*	11	1		*Other predicted functional*		
N-acetylglucosamine*	+	+		Pilin biosynthesis	+	+
glycine*	+	+		Capsule polysaccharide synthesis	+	
cysteine*	+	+		gliding motility		+
glucose	+	+		swimming motility	+	
Phenylacetate*	+	+		bacteriophytochromes	+	+
pectate		+		PhyR (phyllosphere induced regulator)	+	
fucose		+		catalase	+	+
glycerol		+		polyphosphate kinase	+	+
xylan		+				
alginate*		+		*Cell-cell interaction genes*		
galactose	+			HSL-mediated quorum sensing	+	
citrate	+			Putative quorum quenching lactonase YtnP		+
xylose	+			AI-2 transport protein TqsA	+	+
melibiose	+			type IV secretion system	+	
mannitol/fructose	+			RiPP-like bacteriocin	+	
betaine	+			Beta-lactone antimicrobial	+	
glycerol −3-phosphate*	+			T3PKS biosynthesis		+
DMSP* demethylation (DmdA)	+			Lanthipeptide class V synthesis		+
Sialic/Polysialic acid utilization	+			Terpene biosynthesis		+

Based on prokka annotation and COG and AntiSmash analysis [83]. Asterisks indicate compounds found in diatoms [20, 84].

The genomes of SA1 and A30 encode predicted pathways for utilizing numerous organic and inorganic compounds, including those known to be excreted or contained within diatoms (Table 2). Specifically, both genomes encode predicted pathways for using polyamines, *N*-acetylglucosamine, glycine, peptides, cysteine, glucose, and phenylacetate. Interestingly, only strain SA1 encodes DmdA, which is required for demethylation of dimethylsulfonoproponate (DMSP), a metabolite excreted by phytoplankton and used by co-occurring heterotrophic bacteria [55]. SA1 and A30 also encode predicted polyphosphate kinase enzymes, which can break down and use the phosphorous source polyphosphate, also produced by diatoms [56]. Thus, the genomes reveal predicted metabolic strategies consistent with an increase in bacterial cell abundance for strains A30 and SA1 in coculture with the diatom, relative to growth alone in media lacking a source of organic carbon (Fig. 4G).

Finally, both SA1 and A30 genomes encode predicted functional genes that could facilitate the observed physical interaction with diatom cells (Table 2). For example, both strains encode genes predicted to produce pilins, and SA1 also encodes predicted capsule polysaccharide biosynthesis genes. Either pilins or capsule could allow cells to “stick” to the diatom frustule and/or sugar molecules that may be on their cell surface [21].

## Discussion

Our study revealed several successful strategies for concurrent isolation of diatom and closely-associated bacteria from SO water. First, we isolated bacterial representatives from the 3 µm fraction of a SO phytoplankton enrichment culture to favor bacteria that are attached to phytoplankton cells. We chose three phylogenetically diverse isolates obtained from this approach for our coculture experiments: *Pseudoalteromonas* sp. A1, *Olleya* sp. A30, and *Colwellia* sp. A38. Species of these genera have been previously isolated from other polar environments [30, 54, 57, 58]. Moreover, species from the genera *Pseudoalteromonas, Olleya*, and *Colwellia*, have been reported to produce ecologically important exudates, including fatty acids/lipids, antimicrobials, agarolytics, and exopolysaccharides [54, 57, 59, 60] *Olleya* species are well-known degraders and members of the ‘marine clade’ of the *Flavobacteriaceae*, which contribute significantly to the remineralization of organic matter [61]. Of the three isolates we selected, two were found to physically associate with *P. subcurvata* cells in coculture (Fig. 5), validating our method for isolating closely-associated bacterial strains.

Our second approach to isolate a diatom-specific epiphyte was to culture directly from the xenic monoclonal diatom culture that was derived from the initial SO enrichment culture, and therefore carried its bacterial epiphytes through many passages in culture. Using this approach, we cultured two bacterial morphotypes: *Sulfitobacter* sp. SA1, and SA3, which was a mix of *Glaciecola* sp. and *Salegentibacter* sp. Among these cultured representatives, one promoted the growth of *P. subcurvata* (SA1) in the absence of B vitamins and both were seen to physically associate with *P. subcurvata* cells in coculture. *Sulfitobacter* species have previously been isolated from temperate *Pseudo-nitzschia* species [5, 62], and are observed to induce a range of interactions including production of the growth hormone indole-3-acetic acid (auxin) and degradation of DMSP produced by the diatom host. *Glaciecola* species have previously been associated with diatom blooms in cold waters [58]. Thus, these approaches could be used to isolate physically-associated, ecologically-relevant partners from diverse aquatic habitats.

Our data also indicate that the naturally associated bacterial community co-occurring with *P. subcurvata* in the xenic cultures is required for survival in stationary phase (Fig. 3B). Stationary phase can be induced due to nutrient (N, Si or P) limitation in a closed system, such as the one used here. Previous work has shown that, under N-limitation, bacterial addition can reduce diatom mortality, suggesting that bacteria can help remineralize N to prolong diatom survival [63]. While the mechanism by which one or more of the native bacterial species promote *P. subcurvata* survival is not yet known, one possibility is that native bacteria are able to provide *P. subcurvata* with remineralized forms of nitrogen (e.g., ammonium) [63]. Together, these findings indicate that the relationship between bacteria and diatoms is complex, and the roles of each partner likely differ based on environmental conditions and physiological capabilities.

Our concurrent isolation approaches identified two bacterial isolates that differentially affected diatom growth in positive and negative ways (Fig. 4E). In B vitamin deplete cultures, *Sulfitobacter sp*. SA1 increased growth of the diatom compared to the control, suggesting that SA1 supports some of the vitamin requirements of this diatom, even though these cocultures did not achieve the same high growth rate as that of vitamin-replete cultures (Fig. S2). This result aligns with findings that diatom abundance in the SO responds positively to the addition of bacterial-derived B_12_ and Fe [29], and others have reported the addition of B_12_-producing bacteria can enhance phytoplankton growth [13, 30]. Evidence of biotin, thiamine, and B_12_ biosynthesis genes were found in *Sulfitobacter* sp. SA1, supporting our observation that physically attached *Sulfitobacter* sp. SA1 can improve growth of a diatom under vitamin-limiting conditions, even though it appears *Pseudo-nitzschia* species may produce their own biotin [64]. *Sulfitobacter* sp. SA1 may promote diatom growth in other ways. For example, the Antarctic diatom *Amphiprora kufferathii* is found with attached *Sulfitobacter* and *Colwellia* species, and these epiphytic bacteria provided antioxidant functions in the form of catalase activity to promote diatom growth [65]. Our *Sulfitobacter* sp. SA1 isolate encodes catalase (Table 2), and could similarly help *P. subvurvata* detoxify metabolic byproducts, in addition to supplying the diatom with essential vitamins. It is not surprising that *Olleya* sp. A30 negatively impacted diatom growth, given that it lacks the genes to synthesize B_12_, but encodes genes to degrade diatom-derived organic compounds, and was commonly observed to occur within empty frustules, suggesting an ability to invade and consume diatom cells. If *Olleya* sp. A30 cannot synthesize its own B_12_ it would contribute to the uptake of, and competition for B_12_ in the SO; directly competing with diatoms for external sources of vitamin B_12._

In addition to vitamin biosynthesis capabilities, bioinformatics analysis of A30 and SA1 genomes revealed additional predicted functions of ecological significance. For example, both isolates encode predicted bacteriophytochromes, or light-responsive photoreceptors (Table 2). Bacteriophytochromes sense light (usually red or far-red wavelengths), and depending on their N-terminal domain architecture, can mediate various physiological responses by controlling downstream gene expression or enzymatic function [66]. Although it is unknown how these photoreceptors regulate the cellular system for strains SA1 and A30, one prediction is that the bacteriophytochromes could provide a mechanism for these bacteria to coordinate their own physiology and metabolic capabilities with their phototrophic host.

**Fig. 6:**
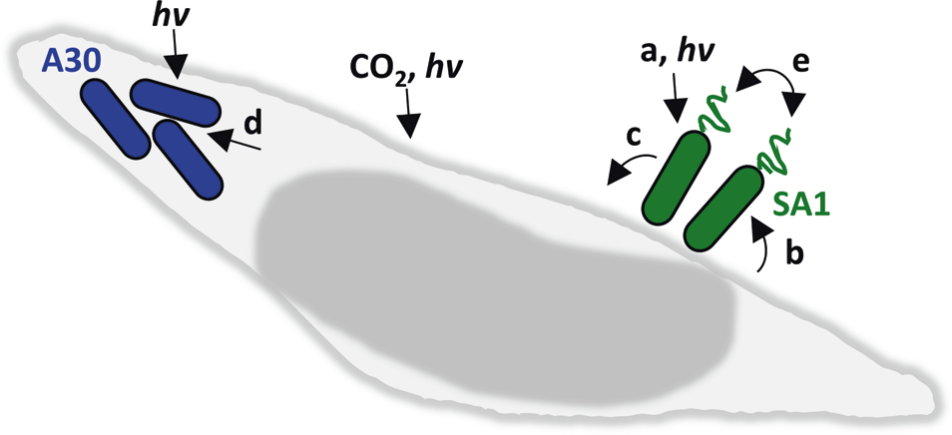
Model summarizing experimental and bioinformatics findings for bacterial-diatom interactions. Diagram shows a single *P. subcurvata* cell with associated *Olleya* sp. A30 (blue) and *Sulfitobacter* sp. SA1 (green). Abbreviations: a iron and other inorganic nutrients; b organic carbon, DMSP polypohosphate; c vitamins, detoxification (ex. catalase), iron, protection; d organic carbon, polyphosphate, iron; e HSL-mediated quorum sensing.

Strains SA1 and A30 also displayed some interesting differences in their functional predictions. For example, while both isolates encode motility mechanisms, SA1 is predicted to use swimming motility while A30 encodes genes suggesting gliding capabilities. Furthermore, they appear to encode different iron acquisition and storage strategies: SA1 encodes distinct predicted iron uptake systems, compared to A30, and SA1 encodes several ferritin-like proteins for possible iron storage (Table 2). Interestingly, SA1 encodes a homolog of PhyR (phylosphere-induced regulator), which was first described in another alphaproteobacterium, *Methylobacterium extorquens* AM1, where it regulates stress response genes and is required for epiphytic growth on its plant host [67]. Moreover, SA1 and A30 encode predicted biosynthetic genes for different small molecules shown to be used in cell-cell interactions and competition in other bacteria (Table 2). For example, SA1 is predicted to make a homoserine lactone (HSL) quorum sensing molecule for interbacterial signaling [68], while A30 encodes a putative quorum quenching lactonase that is predicted to degrade or “silence” HSL quorum sensing signaling [69]. Finally, SA1 and A30 both encode predicted biosynthetic gene clusters for putative antimicrobial molecules, including bacteriocins and beta-lactones, which could be used for interbacterial competition in situ, or as potential new therapeutics [70]. Taken together, these differences in predicted functions suggest that, although SA1 and A30 both are capable of using diatom-derived nutrients for growth (Fig. 4G), they have evolved divergent strategies for motility, cell-cell interactions, and iron acquisition and storage, which limits phytoplankton growth in the SO. In Fig. 6, we summarize our experimental and bioinformatics results to illustrate how the members of this tripartite model system, composed of a diatom and two bacterial isolates, interact with each other and their environment.

Our discovery that a naturally co-occurring growth promoter and degrader physically associate with the diatom host has significant implications for the fate of diatom carbon. For SO diatoms like *P. subcurvata*, which require a biologically derived source of vitamins to thrive, their attached beneficial symbionts (like SA1) can act as extracellular organelles, traveling with the diatom throughout the water column. This work only assayed growth effects under constant light conditions, but given the presence of predicted light-sensing proteins for SA1, it is tempting to hypothesize that SA1 may shift its physiology in response to light quantity and quality, and such a shift could have effects on the host diatom cell. Moreover, if the diatom is no longer able to fix carbon and provide its epibiont with organic carbon, might the nature of its relationship change? Such a shift has been reported in other Roseobacter-phytoplankton interactions where at first the roseobacter promotes phytoplankton growth, only to kill and consume it once the phytoplankton cells begin to senesce [25].

Similar considerations should also be made for degraders, like A30, that can physically associate with the diatom and appear to reside within the cells. The ability to physically contact a host cell would allow degraders like A30 to use extracellular enzymes to degrade not only compounds in the phycosphere, but molecules found inside the cell. Indeed, in our cocultures with A30 and *P. subcurvata*, we did not see the usual intracellular structures (like chloroplasts, Fig. 5C), and the RFU decreased over time with increasing A30 cell abundance (Fig. 4). These results suggest that A30 is able to infiltrate the diatom cell and consume and grow on intracellular content. In support of this finding, a recent study showed that Flavobacteriales (like A30) preferentially consumed ^13^C-labeled diatom lysate, compared to labeled exudate [71], suggesting degraders like A30 may have evolved to fill a niche that utilizes intracellular diatom DOM, either by cell lysis via other organisms or mechanisms, or by directly invading cells.

Such physical associations with diatoms have implications for possible interbacterial cooperation and competition within this microhabitat. At a concentration of 10^6^ cells ml^−1^ in seawater, free-living bacteria are diffuse enough in the water column not to come into physical contact with one another [72]. However, if bacterial cells become associated with a particle, or a diatom cell, competition for space within the phycosphere is highly probable. Indeed, our microscopy images showed instances where bacterial cells were in physical contact on or inside the diatom cell (Fig. 5D, E). For cases where many bacteria of the same strain may crowd together in a low-diffusion microhabitat within the phycosphere or diatom cell, such interactions could facilitate cell-cell communication via HSL signaling molecules like those encoded in SA1. By contrast, touching cells might compete for access to the environment, perhaps by using some of the predicted antimicrobial functions found in the SA1 and A30 genomes. If contact occurs between competing bacterial species, one can imagine that lethal weapons, such as bacteriocins predicted in SA1, could be used to defend the host niche. Indeed, the ability of roseobacters to kill competing bacterial cells has been reported in other isolates from free-living and host-associated habitats [73–79]. The use of genetically modified bacterial symbionts will facilitate tracking and quantifying interbacterial competition in the phycosphere and allow researchers to observe whether these cells are also communicating with each other via bacterial pheromones.

Finally, we considered how this symbiotic association may be impacted by future climate conditions. Climate models predict an increase in sea surface temperature (SST) of 0.3–1.6 °C by 2100 for the Southern Ocean [80], however, the impact of rising temperatures on the SO microbial community is largely unknown. A recent study by Tonelli et al. used machine learning to model predicted effects of future SST on pelagic microbial communities in the SO [80]. The model predicted a decrease in microbial diversity, including a decrease in groups of biogeochemically important bacteria and archaea, which could have cascading effects on ocean chemistry and impact primary production, and thus higher trophic levels. Our isolates were able to grow well at higher temperatures (up to 10–12 °C was tested), suggesting an increase in SST alone is not inhibitory, but it is unknown how the cascading effects described above might impact these attached symbionts. Experiments using SO phytoplankton have shown that tolerance to increasing SST can be influenced by light, Fe, and CO_2_ [81, 82], thus it is likely that bacterial fitness at higher temperature would also impact phytoplankton growth and carbon flux. Future experiments using this co-culture model system could help determine how the mutualistic SA1 or parasitic A30 might impact *P. subcurvata* growth and survival under future climate conditions.

In summary, we were able to successfully isolate diverse co-occurring bacterial strains that form physical attachments to an ecologically-relevant diatom genera in the Southern Ocean that is globally distributed. Our initial characterization of a growth-promoter (*Sulfitobacter* sp. SA1) and a degrader (*Olleya* sp. A30) establishes this interaction as a tractable and informative co-culture model system that can be used to further probe important questions relating to carbon export under current and future climate scenarios.

## Supplementary information

Supplementary information

## Data Availability

All data generated or analyzed during this study are included in this published article and its supplementary information files. Bacterial-genome sequence data are available at the US National Center for Biotechnology Information (NCBI; Bethesda, MD, USA; Accession numbers are listed in Supplemental Table S1).
