# Supplementary material for: A tripartite model system for Southern Ocean diatom-bacterial interactions reveals the coexistence of competing symbiotic strategies
Source: ISME Commun. 2022 Oct 3;2:97. doi: 10.1038/s43705-022-00181-w (PMC9723598; doi:10.1038/s43705-022-00181-w)

Supplemental Table 1. General information about diatom and bacterial strains used in this study

Diatom strain	Species	Collection description<br>Geography	 Ecology	Reference	NCBI Genbank Accession Number for 18S	Morphology
UNC1901	*Pseudonitzchia subcurvata*	West Antarctic Peninsula	Seawater sample collected from Palmer LTER station 200.040	This study	OM993293	Pennate
**Bacterial strain**	**Species**	**Collection description<br>Geography**	**<br>Ecology**	**Reference**	**NCBI Genbank Accession Numbers for draft genome or 16S**	**Isolation Method[a]**
SA1	*Sulfitobacter sp.*	“”	*P. subcurvata*	This study	SAMN25067898*	Monoculture
SA3	*Glaciecola sp.* and *Salegentibacter sp.*	“”	*P. subcurvata*	“”	ON011685 and ON011686	Monoculture
A1	*Pseudoalteromona s sp.*	“”	Seawater sample collected from Palmer LTER station 200.040	“”	ON011687	Enrichment
A30	*Olleya sp.*	“”	“”	“”	SAMN25067897* or ON011688	Enrichment
A38	*Colwellia sp.*	“”	“”	“”	ON011689	Enrichment
F1	Isolate F1	“”	“”	“”	ON011698	Enrichment
E11	Isolate E11	“”	“”	“”	ON011695	Enrichment
E1	Isolate E1	“”	“”	“”	ON011699	Enrichment
E2	Isolate E2	“”	“”	“”	ON011700	Enrichment
I2	Isolate I2	“”	“”	“”	ON011693	Enrichment
A17	Isolate A17	“”	“”	“”	ON011692	Enrichment
E20	Isolate E20	“”	“”	“”	ON011694	Enrichment
A43	Isolate A43	“”	“”	“”	ON011690	Enrichment
E10	Isolate E10	“”	“”	“”	ON011696	Enrichment
A19	Isolate A19	“”	“”	“”	ON011691	Enrichment
F4	Isolate F4	“”	“”	“”	ON011697	enrichment

*Indicates draft genome accession number

[a] Indicates isolation method from *P. subcurvata* monoculture or seawater enrichment sample

Supplemental Figure 1. *P. subcurvata* abundance (RFU) at the end of the coculture experiment (Day 42). Error bars are the standard deviation of 3 biological replicates (n=3). Asterisks indicate statistical significance between values for *P. subcurvata* alone vs with bacterial coculture: One-way ANOVA, Multiple comparisons, **** indicates p<0.0001, ns indicates no significant difference.

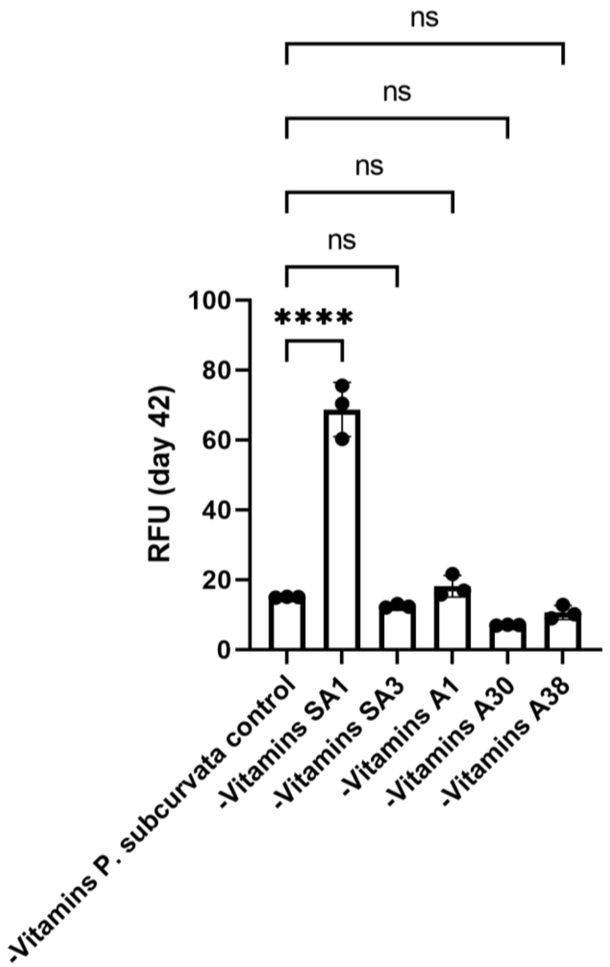

.

Supplemental Figure 2. Specific growth rate of *P. subcurvata* cocultures during exponential phase growth. Growth rate is calculated from the least squares regression of the natural log RFU values of the cultures in the relevant exponential growth period. For the +Vitamins control (*P. subcurvata* alone) treatment, exponential growth occurs between Day 1-7. For both of the -Vitamins treatments: -Vitamins control (*P. subcurvata* alone), and -Vitamins +SA1; the exponential growth period is observed from Day 17 until Day 52. Growth rate of the xenic *P. subcurvata* culture shown in Figure 2C is included for pairwise comparison. Error bars indicate standard deviation. Asterisks indicate statistical significance between values for *P. subcurvata* alone vs with bacterial coculture: 2way ANOVA, uncorrected Fisher's LSD, **** indicates p<0.0001, * indicates p<0.05.

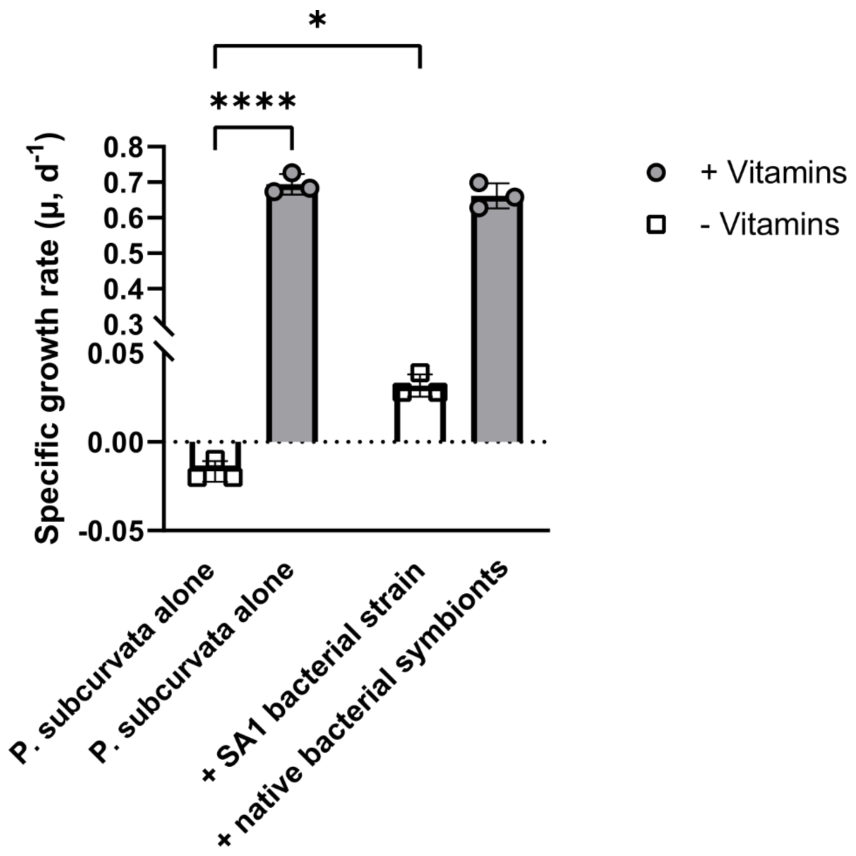

Supplement: Supplementary file 1 — Supplementary information [file 43705_2022_181_MOESM1_ESM.pdf]
